# Review of Classification Systems for Adult Acquired Flatfoot Deformity/Progressive Collapsing Foot Deformity and the Novel Development of the Triple Classification Delinking Instability/Deformity/Reactivity and Foot Type

**DOI:** 10.3390/jcm13040942

**Published:** 2024-02-06

**Authors:** Chandra Seker Pasapula, Makhib Rashid Choudkhuri, Eva R. Gil Monzó, Vivek Dhukaram, Sajid Shariff, Vitālijs Pasterse, Douglas Richie, Tamas Kobezda, Georgios Solomou, Steven Cutts

**Affiliations:** 1 The Queen Elizabeth Hospital Kings Lynn, NHS Foundation Trust, King’s Lynn PE30 4ET, UK; chandras.pasapula@qehkl.nhs.uk (C.S.P.); makhib@doctors.org.uk (M.R.C.); tamas.kobezda@qehkl.nhs.uk (T.K.); 2 Hospital Universitario Doctor Peset, 46017 Valencia, Spain; evaregimo@gmail.com; 3 University Hospitals Coventry & Warwickshire, Coventry CV2 2DX, UK; vivek.dhukaram@uhcw.nhs.uk; 4 Medway Maritime Hospital, NHS Foundation Trust, Kent ME7 5NY, UK; sajid@doctors.org.uk; 5 Riga East Clinical University Hospital, LV-1038 Riga, Latvia; vitalijs.pasters@aslimnica.lv; 6 California School of Podiatric Medicine, Samuel Merritt University, Oakland, CA 94609, USA; drichiejr@aol.com; 7 School of Clinical Medicine, University of Cambridge, Cambridge CB2 2EL, UK; 8 James Paget University Hospitals, NHS Foundation Trust, Great Yarmouth NR31 6LA, UK; stevenfrcs@hotmail.com

**Keywords:** AAFD, PCFD, classification, systematic review

## Abstract

**Background:** Classifications of AAFD/PCFD have evolved with an increased understanding of the pathology involved. A review of classification systems helps identify deficiencies and respective contributions to the evolution in understanding the classification of AAFD/PCFD. **Methods:** Using multiple electronic database searches (Medline, PubMed) and Google search, original papers classifying AAFD/PCFD were identified. Nine original papers were identified that met the inclusion criteria. **Results:** Johnson’s original classification and multiple variants provided a significant leap in understanding and communicating the pathology but remained tibialis posterior tendon-focused. Drawbacks of these classifications include the implication of causality, linearity of progression through stages, an oversimplification of stage 2 deformity, and a failure to understand that multiple tendons react, not just tibialis posterior. Later classifications, such as the PCFD classification, are deformity-centric. Early ligament laxity/instability in normal attitude feet and all stages of cavus feet can present with pain and instability with minor/no deformity. These may not be captured in deformity-based classifications. The authors developed the ‘Triple Classification’ (TC) understanding that primary pathology is a progressive ligament failure/laxity that presents as tendon reactivity, deformity, and painful impingement, variably manifested depending on starting foot morphology. In this classification, starting foot morphology is typed, ligament laxities are staged, and deformity is zoned. **Conclusions:** This review has used identified deficiencies within classification systems for AAFD/PCFD to delink ligament laxity, deformity, and foot type and develop the ‘Triple classification’. Advantages of the TC may include representing foot types with no deformity, defining complex secondary instabilities, delinking foot types, tendon reactivity/ligament instability, and deformity to represent these independently in a new classification system. **Level of Evidence:** Level V.

## 1. Introduction

Adult Acquired Flatfoot Syndrome AAFD had originally become synonymous with the term Posterior tibialis Tendon Dysfunction (PTTD). The original position on AAFD (adult acquired flatfoot deformity) secondary to tibialis dysfunction is almost entirely influenced by the work of Johnson and Strom [1]. In 1989. Johnson and Strom proposed a sequence of stages with progressive failure of the tibialis posterior through the stages, resulting in synovitis, subsequent elongation and tears, and eventual rupture of the tendon in stage 3 deformities. Their classification system, which was both anatomic and clinical, was for the first time able to look at a spectrum of deformities and allow them to be graded and communicated. For almost 20 years, it had been accepted as the standard to which we base our diagnosis and treatment. They presented clinical findings on the state of the foot and then related this to the state of the tibialis posterior tendon, implying its elongation in stage 2 resulted in the characteristic planus deformity. The tibialis posterior tendon was thought to be the primary dynamic stabilizer of the medial longitudinal arch, and its failure/dysfunction results in a cascade of events causing a sequence of structural changes in the foot, with a fixed planovalgus deformity being the endpoint. Further classifications that followed were modifications of this initial classification system. In 1997, Myerson [2] realized that a planus foot may eventually lead to a cyclical deforming force on the ankle, leading to the structural failure of the deep deltoid and a subsequent valgus ankle. Weinraub and Heilala in 2000 [3] were crucially able to delink deformity in the foot with the state of the tendon. They believed that the stage of tendon pathology could exist with or without deformity. However, treatment options still heavily relied on debriding the tendon. They realized that the foot may be rigid and incorporated this into their classification. They were able to target treatment based on the above factors. This classification was a significant advance and often omitted in the evolution of thinking. Bluman in 2007 [4] adapted the Johnson classification to expand and subclassify stage 1 and 2 deformities. Stage 1 tendon deformity largely was based upon the presence of PTT synovitis and then partial tear, and partial tear of the TP with deformity. In stage 2, forefoot deformity was further subclassified into flexible, fixed, and abducted. Treatment options were discussed for the various stages of deformity. In 2008, Deland [5] subclassified the Myerson stage 4 into flexible and fixed, allowing recognition for deltoid reconstruction options for stage 4a but an ankle replacement/fusion for 4b. Parsons in 2010 [6] subclassified stage 2 deformity dependent on the degree of supination deformity with the hindfoot in neutral. Treatment of the forefront deformity was then geared towards doing nothing or correcting the deformity. In 2012, Raikin [7] developed a classification system based on the Johnson classification. The RAM classification breaks the AAFD into the individual components involved in the disease process.

The authors have maintained the grade I–III system and (a) and (b) subclassification currently in use but have applied these separately to the rearfoot (R), ankle (A), and midfoot (M). While the stages of pathology were maintained, the deformity was classified based on deformity location, rearfoot, ankle, and midfoot. In 2017, Pasapula believed that although tibialis synovitis did occur, it was not the main pathology and was a reactive phenomenon that occurred as a result of instability. Stage 0 was added to demonstrate that a high degree of talonavicular laxity was the prime pathology and can occur in the absence of tibialis reactivity but can precipitate the foot to progress into planus as the first ray concomitantly/secondarily fails. A further recognition of the fact that AAFD’s prime pathology was not based upon the tibialis posterior was finally culminated in the deformity based PCFD or Progressive Collapsing Foot Deformity classification. Myerson, in 2020, was able to completely remove the tibialis posterior, rename the pathology and make it deformity focused.

**Figure 1:**
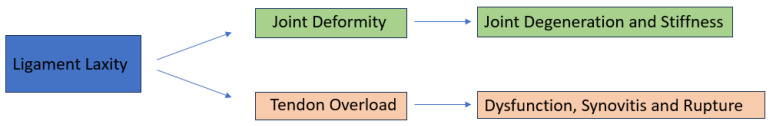
*Clinical components of AAFD/PCFD*.

AAFD/PCFD is an evolving, complex subject. It remains a clinical diagnosis supported by radiological investigations. As shown in Figure 1, the clinical picture comprises a combination of ligament laxity, deformity, reactive tendinopathy, joint stiffness, and degeneration [1,8,9]. Initial starting foot morphology varies significantly, masking or accentuating the clinical picture. Many classifications have emerged, with the most recent focusing on deformity [10].

### Aims

To conduct a narrative review that would help identify advantages and drawbacks of existing classification systems and thus develop a new approach to classifying AAFD/PCFD based on the deficiencies identified.

## 2. Methods

**Figure 2:**
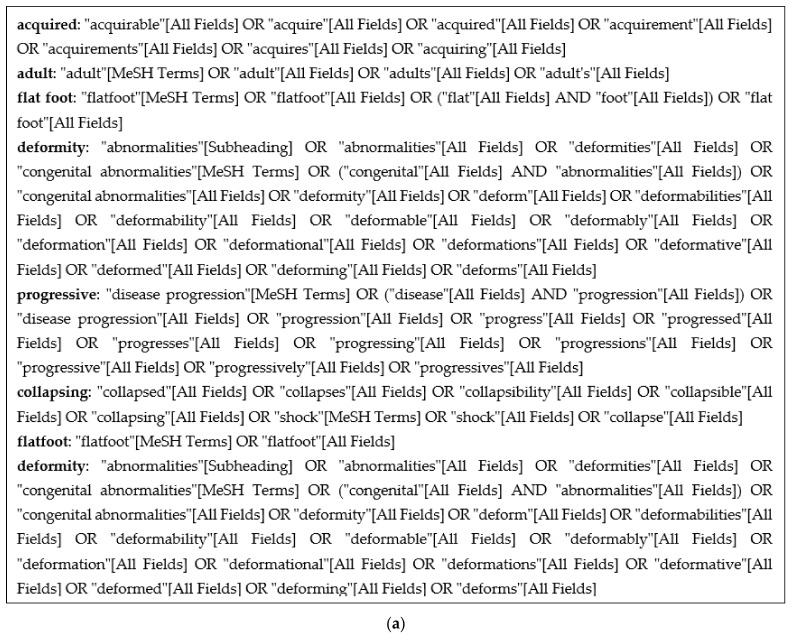

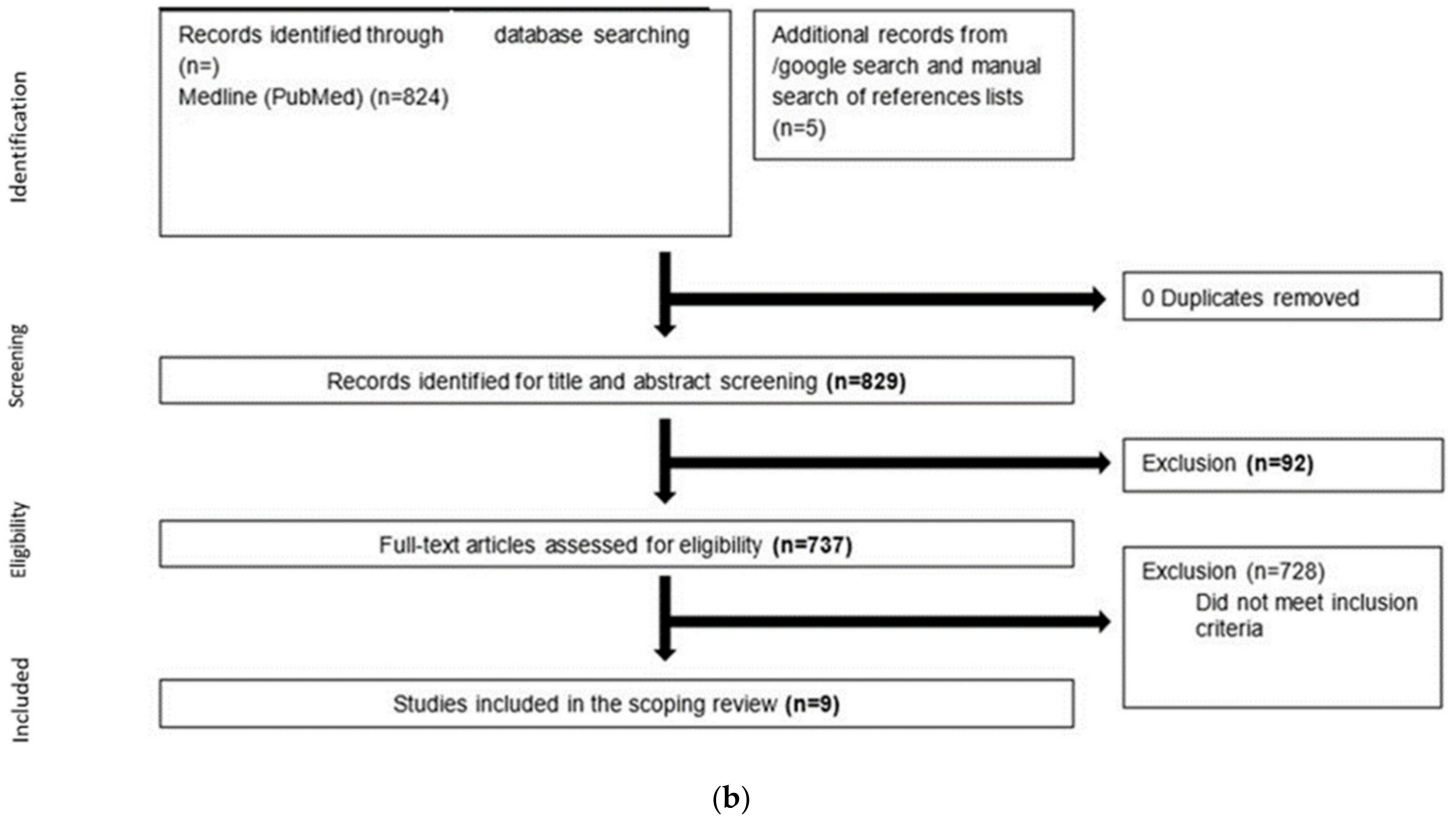
(**a**) *MeSH term strategy.* (**b**) PRISMA diagram showing the selection of papers for inclusion.

The literature search strategy was developed using medical subject headings (MeSH terms) and text words related to ‘flatfoot pathology’, as shown in Figure 2a. Studies were independently identified studies by searching Medline (PubMed interface). The search was conducted on 4 March 2023. A further search of the reference lists from all preliminarily identified papers was also carried out.

The eligibility criteria for inclusion in this scoping review were new classifications for classifying flatfoot, as shown in Figure 2b. Exclusion criteria were publications (i) cadaveric-based studies, (ii) no English translation available, (iii) no human subjects, (iv) studies that were radiographic/MRI descriptors only, and (v) studies where only the abstract is available, as shown in Table 1.

## 3. Results

### A Critical Review of Classifications

The initial understanding of AAFD/PCFD was based on Johnson’s Classification (1989) [1], which used clinical findings to communicate pathology. It erroneously assumed that primary synovitis/tears of the tibialis posterior (TP) tendon (Johnson stage 1) were causal rather than reactive, resulting in weakening of the medial arch, progressive collapse (Johnson stage 2), and fixed deformity (Johnson stage 3) [1]. Clinical, cadaver, and computational modelling studies demonstrate that TP subtraction does not necessarily result in progression to unstable planovalgus (in AAFD/PCFD) [12,13,14,15,16,17]. Finite element foot modelling demonstrates peroneal longus (PL) and TP tendon overload with simulated spring ligament (SL) laxity, reinforcing that tendon pain is most likely reactive and not causal. The Johnson classification implies the foot starts in a neutral posture and progresses to planus, yet many feet have pre-existing painless contralateral feet [18]. Controversy exists around the presence of Johnson’s stage 1 AAFD/PCFD [10]. This stage represents a crucial stage in some feet’s natural history, alluding to the presence of midfoot ligament instability [SL/superficial deltoid] with a stable first ray that resists planus [1,10,11]. Its identification presents an opportunity to intervene early, before the onset of first ray instability (FRI) and complex stage 2 deformities.

Initial classifications [1,3,4,11] implied progression between stages, which later, classifications removed [10]. Progression rates between stages are undetermined, and feet may not present at a specific stage or progress through all stages. Many feet are treated successfully with orthotics, remaining static if tendon reactivity can be persuaded to settle.

Johnson’s classification and its variants incorporated aspects of instability and stiffness [1,3,11]. Later classifications, including Bluman’s classification used weight-bearing (WB) radiographs to account for talonavicular (TN) joint uncoverage [4], recognizing the SL’s role in stage 2 pathology but continued to focus on the TP tendon. Deltoid abnormalities were brought in later foot classifications in stage 3 [4] and into the RAM classification [7]. Myerson added to stage 4 to recognize that deep deltoid rupture results in ankle valgus [2], further subclassified by Deland in 2008 [5]. Bluman in 2007 further expanded stage 2 to differentiate flexible and fixed first ray deformity and the extent of talonavicular uncoverage but continued to focus heavily on the state of the tibialis posterior tendon [4].

Based on deformity location, the RAM classification implied that the TP tendon was the starting pathology in their stage 1 Rearfoot classification. Attempts to subclassify Johnson’s stage 2 based on the degree of forefoot supination were made by Parsons et al. in the Truro classification [6]. Later, stage 0 was introduced [11] to define a stage of Talonavicular/spring ligament laxity and reaffirm its ligament’s role as the primary precipitating pathology and not the tibialis posterior, clinically identifiable prior to first ray dorsiflexion failure. They believed this was the essential lesion in AAFD/PCFD development. Erroneously, many of these secondary classifications remained ‘deformity-centric’, and although referenced laxity/instability, continued to focus on the posterior tibialis, particularly in the early stages.

In 2020 [10], Myerson proposed a new classification based on joint flexibility/rigidity and deformity location, as shown in Table 2. The consensus group of nine mainly North American surgeons renamed the condition “Progressive Collapsing Foot Deformity” (PCFD). Some aspects failed to reach 100% concordance.

Depending on associated clinical and radiograph findings, the system assesses deformity location and joint flexibility as stage I and rigidity as stage II.

Benefits and drawbacks of classification systems with a summary in Table 3:

**Johnson and Strom’s Classification:**

**Advantages:**

The first attempt at classifying the pathology and developed sequential stages of the pathology. For the first time, communicating the pathology became possible. This became the ‘gold standard that subsequent classifications were based upon.’

**Disadvantages**:

There were several erroneous assumptions. The classification heavily focuses on the tibialis posterior tendon as the prime driver of the pathology. This became a misinterpretation in the understanding and staging of the pathology for two decades. The classification oversimplifies stage 2, which is a complex range of pathologies that were not fully described. There was an assumption that the foot started in neutral; however, Dyal was able to show several patients feet started in planus and became painful subsequently [18]. Several instabilities developed before the rigidity of stage 3. These include subtalar instability, lateral column instability, and medial column instability that were simply not defined or understood. The classification mistakenly assumed that there was no instability in stage 1 and that the tendon became spontaneously inflamed. There has been no explanation for non-synovitic lateral plane instability of the talonavicular axis. The linearity of their progression has never been proven.

**Myerson stage 4**

**Advantages:**

The introduction of stage 4 recognizes the gradual failure of the deep deltoid.

**Disadvantages:**

The failure of the Myerson stage 4 addition to the classification would be the incorporation of the early erroneous stages of Johnson and Strom’s classification into the fourth stage of the flatfoot model.

It fails to consider prior development of anteromedial instability of the ankle also most likely due to deep deltoid attenuation prior to the complete failure of the deep deltoid. We suspect that there is a differential failure of the deltoid ligament initially due to rotational forces transmitted to the talus secondary to medial column laxity. Afterwards, there is valgus failure/complete rupture of deep deltoid of the ligament due to additional valgus forces exerted by the calcaneum at heel strike.

**Weinrub and Heilala**

**Advantages:**

Introduces rigidity and deformity into the classification. It attempted to delink tendon reactivity and deformity. Treatment options were given based on clinical findings.

**Disadvantages:**

The classification still erroneously focuses on the tibialis posterior and erroneously classifies stages of tibialis posterior pathology. There are continued broad links to the Johnson classification. There is no mention of where the deformity is based/localizes. The extent of deformity is not graded. Fixed deformity is not graded. The classification continues to imply that the foot starts in neutral. There is a subjective differentiation of stage 2 and 3 insufficiency based on failure of soft tissues. Soft tissue component failure, such as ligament failure and tendon reactivity, have been grouped into a single group. The classification does not address the first ray instability present with hindfoot valgus. The classification implies that hindfoot valgus is the main driving factor.

**Bluman**

**Advantages:**

Advantages of the Bluman classification includes the significant expansion of Johnson’s original classification, especially in stage 1 and 2.

**Disadvantages:**

Classification still tends to imply that the tibialis posterior is the driver of the pathology, but heavily remains broadly based on Johnson’s classification. The classification does not delink tendon pathology and deformity in the early stages. The tibialis posterior was classified based on presumptive morphology and elongation (this has never been further quantified), making it subjective to interpretation. In stage 1, there is still a presumption that the foot starts in neutral and develops valgus. A main feature of stage 2 is hindfoot valgus, however, this is often exacerbated by secondary first ray instability (not described in the early stages). Generally, the earlier stages focus on the tendon, and the latter stages focus on deformity, however, both these facets may not be present in AAFD/PCFD pathology.

**Parsons**

**Advantages:**

Expands stage 2 to demonstrates multiple types of supination deformities in the forefoot.

**Disadvantages:**

The classification is still based on Johnson and Strom’s classification with all associated flaws. It states that stage 1 is an undeformed foot with pain but lacks sufficient explanation why an undeformed foot progresses to planus. The classification maintains stage 2 pathology primarily arises secondary to tibialis posterior dysfunction. The classification does not describe the spring ligament as involved. Using 15 degrees as a cutoff may be flawed, unproven and subjective, given many feet start in planus, with progressive ligament instability developing.

**RAM classification**

**Advantages:**

Raikin proposed a newer complex classification system in 2012. A main reason was the lack of consideration in the previous classification for the midfoot. It is classified based on zoning deformity and then with subgroups based on clinical and radiographic findings. Deltoid instability and spring ligament deficiencies were recognized in their classification. Treatment algorithms were based on these findings.

**Disadvantages:**

Stage 1 still heavily focuses on the tendon without determining instability or a reason for the instability/reactivity. There is a presumption that the foot starts in neutral.

Several subclassifications and variations in the interpretation of each stage make this difficult to understand and communicate. Feet do not necessarily neatly fit into this picture.

Tibionavicular uncoverage is significantly used, but static radiographs are not reflective of true instability at this joint. Several instabilities, such as lateral column instability, are not described.

Inter-observer reliability is not proven across several studies.

**Pasapula**

**Advantages:**

Conceptually tried to place midfoot SL laxity as central to the pathogenesis of the planus foot, to attempt to remove the tibialis posterior as causal and give a plausible explanation for stage 1 pathology. The introduction of stage 0 pathology presented an advancement, and recognized midfoot instability in the absence of reactive TP.

**Disadvantages:**

Old nomenclature was still used in subsequent staging.

Therefore, subsequent stages have all the disadvantages of the existing nomenclature.

**Myerson**

**Advantages:**

Myerson’s 2020 classification benefits from acknowledging that TP tendon reactivity is not the primary pathogenesis by removing it from its nomenclature. It removes the assumption of linearity of progression that Johnson’s classification makes and the assumption that the foot starts in a normal attitude and progresses into planus through defined stages. The panel did not reach 100% consensus on using advanced modalities in the classification. It is primarily focused on classifying clinical deformity.

**Disadvantages:**

Drawbacks of this PCFD classification remain. Static weight-bearing imaging may miss and can underestimate dynamic instabilities and associated ligament laxity severity, which requires clinical evaluation. Multiple tendon reactivity may be a significant presentation and can present without deformity in the early stages of standard feet and any stage of collapsing cavus feet. As both ‘deformity’ and ‘tendon reactivity’ are secondary manifestations of progressive ligament laxities, both need representation, not just deformity. Subtle congenital and developmental deformities may also further contribute to ligament laxity. Developmental malformation of the anteromedial facet of the subtalar joint may allow subtalar subluxation to develop more easily. Focusing on deformity detracts from representing feet with ligament instability in the absence of tendon reactivity or deformities yet may still progressively collapse. Two hundred forty-two subtypes make a comparative analysis and communication of different grades and stages difficult [19].

Further limitations of PCFD classification may arise from its reproducibility. There is good intra-observer reliability (Cohen kappa = 0.851, *p* < 0.001, 95% CI 0.777–0.926); however, the inter-observer reliability drops to moderate with a kappa value of (Fleiss kappa = 0.561, *p* < 0.001, 95% CI 0.528–0.594) [19,20]. Some PCFD subtypes only had a reliability of 0.07/slight (class C) [19]. Collapsing planus feet constitute most and will be more expressive in their deformity [18]. Deformity-based classifications have an inherent bias in identifying deformity in planus feet with progressive instability. If a cohort of collapsing cavus feet is assessed, the classification may lack sensitivity in detecting deformity/pathology, as these feet would naturally express deformity less. The degree of stiffness prior to becoming a fixed deformity is difficult to represent in classification systems. Li showed that the PCFD classification was not affected by the grade of the surgeon, but some aspects, such as peri talar subluxation, had a 26% misdiagnosis rate [21]. The abbreviations used are also not relatable [22]. Only nine surgeons were ultimately involved. Broadening the consensus with wider consultation of the professional field for future iterations of the PCFD classification may provide further insights and incorporate differential views [22].

## 4. Discussion

### 4.1. Understanding the Origin and Identifying Problems of AAFD/PCFD Classifications

- Deformity in AAFD/PCFD is a variable expression of pre-existing foot posture and progressive instability arising from progressive ligament incompetence.
- Anteromedial deltoid instability, lateral column instability, and significant subtalar instability/subluxation from interosseous ligament failure need representation.
- Overload reactivity of the plantar fascia [11] and the musculotendinous units [23] arise as a result of instability and changes to the subtalar axis (see below). Tendon overload and reactivity vary as the deformity progresses (PL and Tendoachilles), as they become offloaded and may not manifest in all AAFD/PCFD stages.
- Early foot stages with isolated SL laxity (stage 0)] [11] and FRI express no deformity, but the foot may feel unstable and thus need defining and representation. Associated tendon reactivity has been defined as stage 1 [1].
- Cavus foot types with SL laxity and FRI may have no visible deformity yet have significant instability from ligament laxity and tendon [TP and PL] overload pain [23].
- WB [axial gravitational force] stress joints in the axial plane. Many joints act perpendicular to the axial plane and, therefore, may not be expressive of the respective joint instability on weight-bearing radiographs. Joints whose motion acts perpendicular to the axial weight-bearing axis accentuate instability when forces are applied in the direction of their action. (TN joint: lateral plane/ankle: anteroposterior motion instability and rotational ankle instability at the deltoid).
- Foot abduction stress radiographs exacerbate TN uncoverage, and ankle valgus stress views may accentuate deltoid instability. Both may be significantly underrepresented on weight-bearing radiographs.

### 4.2. Key Aspects to Take into Consideration in Any New Classification

#### 4.2.1. Importance of Plantar Fascia (PF) in Protecting the SL and Effects of Tight TA

An intact PF protects SL strain development [14]. Huang showed that the plantar fascia confers a 56% relative contribution to arch stability [24]. Crary [14] demonstrated that plantar fascia sectioning and cyclical cadaver foot loading lead to SL and long plantar ligament (LPL) strain and planus. PF is not represented in AAFD/PCFD classification systems, but its role needs to be discussed. Non-rheumatological plantar fasciitis (NRPF) represents a potential early warning sign of foot progressive instability. SL/TN laxity has been shown in feet with NRPF, with ultrasound studies demonstrating SL thinning in feet with NRPF [25]. Symptomatic NRPF manifests as a reactive tensile overload of the PF [11]. Studies show that in NRPF, altered radiographic foot alignment is present at the TN and subtalar joint articulations [26] along with significant TN laxity, which reflects the short-term therapeutic effect of steroid injections in RCTs (3 months) and failure to address persistent biomechanical tensile overload [27].

Tendon Achilles/gastrocnemius tightness is the primary deforming factor or may develop secondarily to the chronic valgus heel. Its presence is significant as a potential contributor to midfoot laxity. Tightness causes an early heel rise, prolonging inferomedial talar head pressure on the SL, causing strain and a midfoot break. Significant hindfoot valgus and internal rotation of the subtalar axis alters the TA pull and the heel strike ground reaction force vector to act laterally to the subtalar axis to augment hindfoot valgus. TA tightness prevents the reduction of the hindfoot to the neutral axis with ankle dorsiflexion.

#### 4.2.2. The Role of Musculotendinous Units and Their Overload

Musculotendinous units decrease foot ligament stress [protective effect] [1], compensate for ligament failure, and play a dynamic role in contributing to foot arch stability [28]. Cadaver studies demonstrate increased subtalar joint internal rotation without the TP and the plantar fascia and the SL [1]. Their loss does not necessarily lead to planus. Progressive ligament instability and deformity with biomechanical overload differentially overload tendons at different stages of deformity progression, which then act outside their physiological limit to manifest as reactive tendon pain (mainly medial retro-malleolar pain). EMG changes in intrinsic foot muscles, such as the abductor digiti minimi, and extrinsics, such as the peroneus brevis, have been recorded. Increased activity in the PL tendon [29], TP tendon, and Achilles have been noted in collapsing planovalgus feet [30], reinforced by computational modelling [23].

#### 4.2.3. Ligament Laxity

AAFD/PCFD classifications are largely ‘deformity centric’, despite progressive ligament laxity [primary pathology] manifesting as deformity, tendon reactivity, and joint stiffness/degeneration. Determining the sequence of progressive ligament instability that develops in AAFD is important. Deformity as an expression of laxity/instability depends on starting foot morphology, the degree of ligament laxity, joint stiffness/degeneration, and the amount of axial load applied in the context of pain.

##### What Is New about Instability/Ligament Laxity?

1. Progressive ligament laxity is key to symptoms.
2. Progressively collapse manifests as soft tissue reactivity and deformity, varying between individuals.
3. Feet may not progress through all instability stages. Instability starts medially at the spring ligament and its associated superficial deltoid attachment (deltospring ligament complex) and commonly associated secondary first ray instability to progress to more widespread laxity involving the subtalar joint, ankle, and the lateral column and the ankle. Progression rates may vary.
4. Some feet start with pre-existing laxity that has been physiologically normal for that foot, thus not painful. Increased instability progresses the foot to become symptomatic. Normal laxity for any foot may be gauged by contralateral foot comparison if unaffected, from serial foot assessment, or may never be ascertained if no pre-existing reference point exists.
5. Lateral column instability, subtalar instability, and anteromedial deltoid instability reflect a greater extent of foot ligament failure than the isolated failure of the medial column. Addressing the medial column alone (superficial deltoid/spring and first ray) may not restore all the foot instabilities that have developed completely.

##### Evidence for Sequential/Progressive Instabilities in AAFD

Evidence for sequential instability comes from studies in several disciplines (cadaveric, computational, and clinical). Although multiple ligaments eventually progressively fail, the hallmark of AAFD requires some degree of SL component strain/dysfunction of the wider superficial deltoid–spring ligament complex, which would then allow for inferomedial talar head subluxation and secondary/concomitant first ray destabilization. Using a cadaver model, Jennings created deformities associated with AAFD in a 3-dimensional custom-loaded frame [31]. Significant rotational changes of the talus, navicular, and calcaneus occurred after SL sectioning, which the loaded TP tendon could not restore, despite incremental tensioning [32]. A functioning PF [14] acts as a tie beam for the medial longitudinal arch, and an intact superficial deltoid suspends the SL from the fixed medial malleolus. Their dysfunctions may be difficult to determine but increase SL stress, leading to its strain. Early SL strain, not visible on WB radiographs, needs clinical evaluation [33]. The TN portion of the superficial deltoid also restrains TN abduction [34], and the tibiospring ligament suspends the SL from the medial malleolus thus influencing its function.

There is a potential dichotomy of views regarding the superficial deltoid’s structure with the tibiotalocalcaneal ligament consistently present. Although dense condensations form individual ligament bands/fascicles [31] with variable presence [31], Amaha demonstrated that the internal morphology of the deltoid–spring ligament forms a single continuous structure attached at the medial malleolus [35], thus interlinked and interdependent functionally.

##### First Ray Instability and Its Classification

FRI develops concomitantly or after SL laxity [36], progressing the foot to an unstable planus [12]. The stable first ray exerts an opposing supinating ground reaction force vector that resists foot pronation/inferomedial talar head subluxation with SL strain. The first ray eventually fails in dorsiflexion (failure of the plantar TMT ligaments and/or the plantar NC ligaments). Cyclical loading cadaver studies demonstrate a decrease in Meary’s axis after SL/PF sectioning [36]. Deep deltoid rupture in ankle fractures demonstrates SL weakening [37] and Type 1 FRI (secondary to SL laxity, not hallux valgus) that develops within six months post-injury. Radiographic change in Meary’s axis of 30 degrees has a high (100%) sensitivity for predicting intraoperative SL tears [32].

##### Lateral Column Instability/Laxity

Lateral column instability develops in AAFD/PCFD but is often not documented [14]. The mean increase in lateral column motion in feet with symptomatic AAFD/PCFD is 5.5 mm [38]. SL laxity internally rotates the subtalar axis, lateralizing forefoot load with respect to the subtalar axis at toe-off and therefore contributory. Medial column instability and gait alterations from a painful 2nd MTP joint further transfer load laterally to the middle and lateral foot columns, straining the long plantar ligament and the plantar capsular ligaments.

##### Deltoid Ligament Laxity

SL laxity affects superficial deltoid function (TN/tibiocalcaneal ligaments), and vice versa. Abnormal talar kinematics likely allows stress to transfer to the deep deltoid portion [1]. Forces generated from excessive valgus heel exacerbates talar head abduction, straining elements of the superficial deltoid (tibioavicular/tibiocalcaneal). This further contributes to deep deltoid strain and the development of anteromedial ankle laxity in AAFD/PCFD, not represented in some classifications. Anteromedial ankle instability develops prior to deltoid rupture with ankle capsular failure/valgus ankle (Myerson’s addition of stage 4 to Johnson’s classification). The gravitational external test and the external heel rotation test have been considered the gold standard [39,40] for detecting deep deltoid instability, prior to Johnson’s stage 4 failure. Anteromedial gutter pain sensitivity is unknown as a sign of deep deltoid insufficiency in chronic anteromedial instability. The presence of anteromedial ankle laxity in AAFD/PCFD may change surgical management and may be a persistent cause of PTT overload, which acts to counteract this laxity. This would be akin to peroneal pain with ankle ligament laxity. In our practice, patients with AAFD/PCFD always have a positive lateral push test and often have a positive anteromedial draw test, but sometimes have an external heel rotation test [40], suggesting multiple types of deltoid–spring failures that have yet to be classified.

##### Subtalar Ligament Laxity

The SL acts as a primary restraint to hindfoot valgus. TN joint unlocking, with SL laxity and secondary first ray instability, allows for non-physiological hindfoot valgus. Talonavicular joint laxity [SL laxity] and associated non-physiological heel valgus thrust negatively exacerbate each other with cyclical foot loading. Eventually, subtalar (interosseous) ligaments will strain with hindfoot valgus [41], allowing subtalar instability. SL reconstruction may restore the TN joint axis but may not restore subtalar joint stability or the subtalar axis if subtalar ligaments are compromised. Persistent subtalar instability and impingement (sinus tarsi and fibula) can still exist. Clinical examination aids diagnosis [33]. Sub-fibular/sinus tarsi impingement on WB radiographs and MRI subtalar ligament changes alludes to interosseous ligament instability [41].

Recently, WB CT has been used to evaluate the presence of middle facet subluxation and quantify its uncoverage [8,9] and measure foot and ankle offset. The diagnosis of PCFD was often made clinically. We believe that subtalar capsular failure would not occur without the medial column failing first. The use of WB CT in early spring ligament laxity is unknown. Although the presence of 28.7% middle facet subluxation had 100% sensitivity in diagnosing PCFD, the true correlation of this with actual subtalar instability is not known [8].

Subtalar fusion as part of the treatment may restore stability and limit pain in this scenario. A good reference clinical test examination for subtalar/interosseous ligament instability assessment prior to lateral impingement after talonavicular reduction is still lacking [42].

### 4.3. Foot Type and Potential Differential Behaviour of Cavus Feet

Foot morphology is important. More planus feet destabilize/collapse, given their biomechanical disadvantage [18]. Asymptomatic feet can start with normal, planus, or cavus attitude and varying degrees of intrinsic stability [17]. Scenarios with less deformity expression include collapsing cavus feet and feet with a neutral foot attitude with SL laxity and first ray stability (early stages). FRI may not be radiographically expressed, given the starting plantarflexed position in cavus feet, yet its instability may act to overload the PLT and TP tendon. Deformity-based classifications may struggle to classify AAFD/PCFD in these scenarios.

### 4.4. Triple Classification: Foot Type/Stage of Ligament Laxity/Zone of Deformity

The ‘Triple classification (TC)’ attempts to delink instability, which leads to deformity and degeneration in different foot types. In a highly evolving subject, addressing these entities independently enables a greater representation of stages and types of AAFD/PCFD.

**Figure 3:**
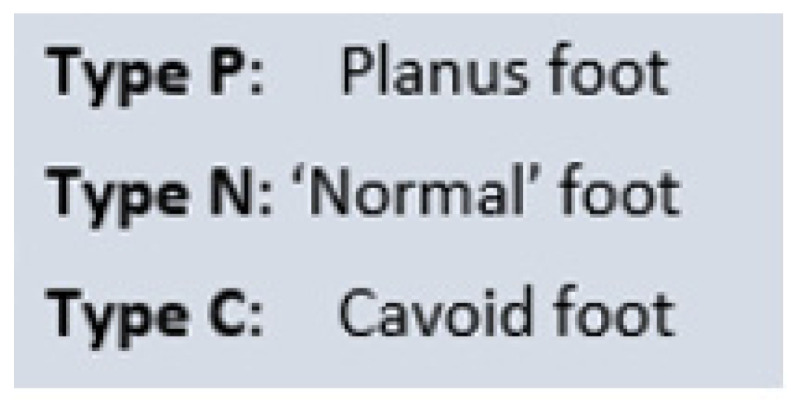
Triple classification foot types.

The ‘backbone’ of the TC is primarily staging feet, as shown in Figure 3, based on progressive ligament laxities. Understanding deformity, degeneration, and tendon/fascia overload are secondary consequences that are variably expressed. The secondary deformity can help anatomically localize ligament laxities clinically manifested as instabilities. Early TC stages may have laxity with no deformity dependent on foot type; later stages have more deformity, rigidity, and degeneration, which can be zoned based on axial radiographs. Flexible stages have laxities and deformities. Rigidity masks prior stages of ligament laxities that have developed.

#### 4.4.1. TC Foot Type

Feet are types based on clinical morphology. Feet can be planus, normal morphology or cavus. All these foot types can progressively collapse, to cause planus symptoms but all collapsing feet do not exhibit deformity.

#### 4.4.2. TC Stage Based on Ligament Laxity/Instability

**Figure 4:**
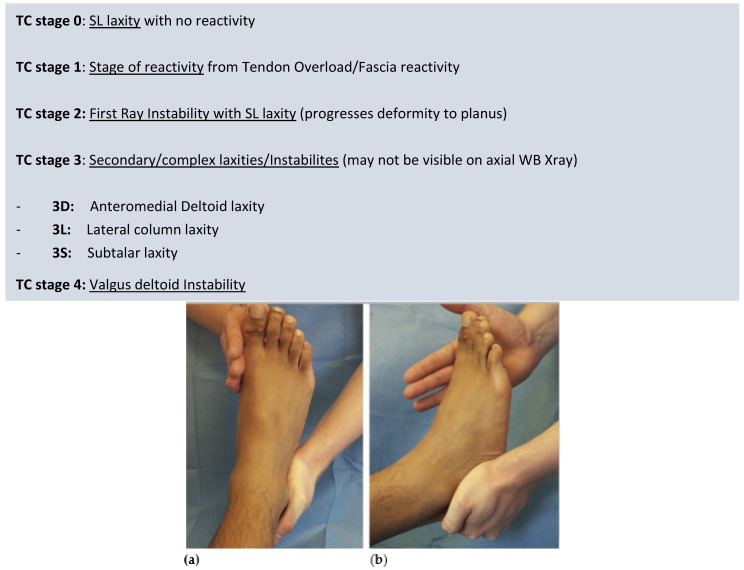
Neutral heel lateral push test. (**a**) dorsal view (**b**) sagittal view.

Progressive ligament instabilities occur combined or discreetly through stages, as shown in Figure 4.

##### Triple Classification Stages

TC stage 0: Loss of SL integrity leads to TN abduction laxity (see above), allowing the potential for foot progression into planus (and development of secondary instabilities). SL integrity loss may arise primarily or due to superficial deltoid (suspends SL) or plantar fascia integrity loss (protects SL). Assessing strain in these two latter structures may be more difficult to clinically ascertain. First ray stability resists planus, and thus crucially planus may not present on examination in feet. SL laxity/TN laxity is the earliest isolated lesion that can be clinically identified in a previously stable foot. Neutral Heel lateral push test (NHLT) is positive with a stable first ray.

TC stage 1: Reactive phase of the foot. Instability with biomechanical overload may cause tendons and fascial reactivity prior to visible planus/deformity and is an important sign the foot is collapsing. SL laxity/TN laxity predisposes the foot to progressive collapse, but the stable first ray prevents planus. We believe the PF reactivity may represent an early warning sign. However, this stage of foot reactivity may not be present in all feet with progressive collapse. A tender reactive TP/Peroneus longus tendon (occasionally) and/or plantar fascia can allude to the presence of underlying TN instability prior to first ray instability.

TC stage 2: First ray dorsal sagittal failure (TMT commonly and/or NC joints) secondary to SL laxity (type 1 FRI) is the hallmark of stage 2 pathology. Stability in this acts as a secondary stabilizer to planus. FRI with a lax SL/unlocking of the TN joint progresses the foot into the planus (a common stage/clinical scenario seen). NRPF commonly seen in this stage, reflecting tensile overload of the plantar fascia.

SL instability: Positive NHLT.

FRI/dorsiflexion (Roots maneuver, Morton’s test, Double dorsiflexion test).

TC stage 3: Secondary foot complex foot instabilities are present. Foot instability is no longer isolated to the TN joint and the first ray (medial column) but begins to demonstrate more widespread instability at the ankle joint, the lateral column, and/or the subtalar joint. Anteromedial ankle instability is secondary to superficial and deep deltoid failure, lateral column instability due to LPL strain [14], and subtalar instability from interosseous strain. These instabilities represent more widespread foot ligament failure/involvement. Instabilities that have arisen beyond the medial column (SL and first ray inability) are explicitly stated, e.g., Stage 3 D (deltoid) or Stage 3DS (deltoid and subtalar).

[L] Lateral column ballottement compared to the contralateral side.

[D] Anteromedial ankle draw test for deep deltoid laxity.

Heel external rotation test for deep deltoid instability.

[S] Modified anterior draw for subtalar instability/other stress tests.

Any combination of these instabilities is possible.

TC stage 4: Deep deltoid failure with capsular failure. The ankle progresses into the valgus. The assessment of instability is clinical and radiographic. Deformity helps anatomical localization of ligament deficits.

**Figure 5:**
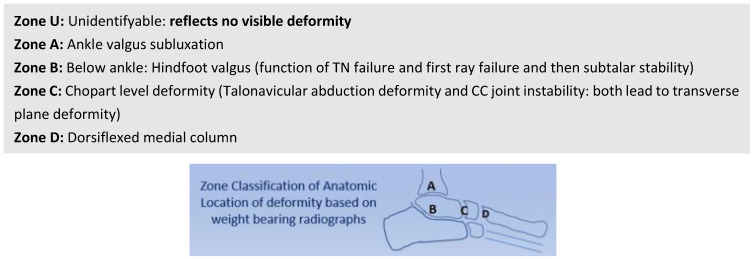
Zonal classification based on the anatomic location of the deformity.

Zoning deformity based on axial stress (weight-bearing) radiographs further aids deformity localization in stages 2, 3, 4 and alludes to foot type, as shown in Figure 5. Early stages of Type N and multiple stages of Type C feet may express no deformity and are classified as Zone U (deformity unidentified).

The role of advanced imaging modalities such as US, MRI, wb CT, and stress views in staging ligament failure may be used but have not been incorporated in this iteration.

The deformity can be further subtyped into r and d subtypes (rigid or with degenerative arthritis) within different zones where there may be a rigid hindfoot but a flexible first ray, and vice versa.

### 4.5. Overview Diagram of the Triple Classification System

**Figure 6:**
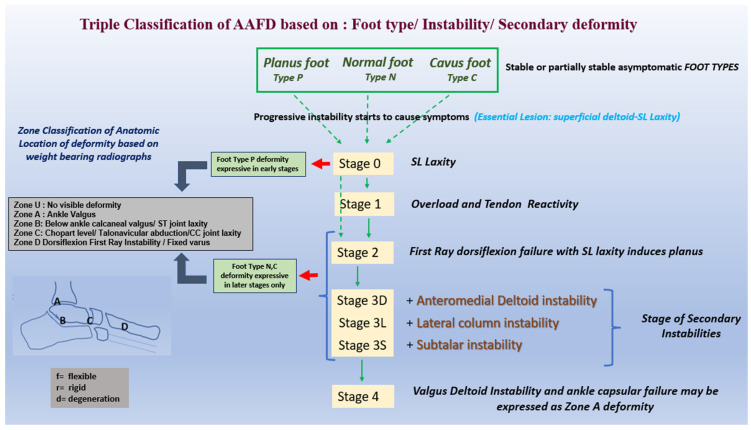
Triple classification of AAFD.

Based on everything we have discussed, feet can be classified based on Foot type, Stages of ligament instability, and Zones of deformity, as shown in Figure 6.

## 5. Limitation

Limitations to the TC system includes several different combinations, despite making subtypes more relatable, may still make communication difficult. Feet with congenital deformities are difficult to represent despite potential ligament laxity. There may be subjectivity in identifying original normal and cavus foot types, particularly with progressive collapse. Future work assessing intra-observer and inter-observer reliability is needed. The classification is focused on identifying subtypes, but future literature may further focus on linking classification to management. The TC system does not address initial causality of instability or determine temporal relationships between stages.

## 6. Conclusions

AAFD/PCFD remains a clinical diagnosis with imaging modalities serving to support augment diagnosis. The fundamental pathology remains a sequential/combined failure of foot ligaments that starts in the medial superficial deltoid–spring ligament complex, whose function is significantly influenced by plantar fascia integrity. Deformity assessment alone may not accurately diagnose stage 0 or complex stage 3 ligament instabilities present in AAFD/PCFD [Triple classification] feet and requires clinical and radiographic assessment. The TC allows feet to be typed, deformity zoned, and ligament laxity to be staged. It also serves as a new iteration that aims to improve clinical assessment, represent more complex instabilities, and aims to make subtypes more relatable thus aiding communication.

## Figures and Tables

**Table 1:** Results.

	Classification and Year	Article	Categories
1	Johnson and Strom [1]	1989	Original stage 1–3 classification
2	Myerson et al. [2]	1997	Myerson modification of stages 1–4
3	Weinraub and Heilala [3]	2002	Stage 1–3 and grades (A, B, C)
4	Bluman et al. [4]	2007	Stage 1–4 (A, B, C subtypes)
5	Deland [5]	2008	Stages 1–4 (7 subtypes)
6	Parsons et al. [6]	2010	Stage 1–4 Stage 2 subtypes (A, B, C)
7	Raikin et al. [7]	2012	RAM classification Three categories and six subtypes
8	Pasapula et al. [11]	2017	Stage 0–4
9	Myerson et al. [10]	2020	Stages 1–2 (5 stages and 2 deformities)

**Table 2:** Myerson’s classification of progressive collapsing foot deformity (PCFD).

Stage of Deformity		
Stage I (Flexible) or Stage II (Rigid)		
Type of Deformity		
	Deformity Type/Location	Clinical/Radiographic Findings
**Class A**	Hindfoot valgus deformity	Hindfoot valgus alignment Increased hindfoot moment arm, hindfoot alignment ankle, foot and ankle offset
**Class B**	Midfoot/forefoot abduction deformity	Decreased talar head coverage Increased talonavicular coverage angle Presence of sinus tarsi impingement
**Class C**	Forefoot varus deformity/medial column instability	Increased talus-first metatarsal angle Plantar gapping first tarsometatarsal (TMT) joint/naviculocuneiform (NC) joints Clinical forefoot varus
**Class D**	Peritalar subluxation/dislocation	Significant subtalar joint subluxation/sub-fibular impingement
**Class E**	Ankle instability	Valgus tilting of the ankle joint

**Table 3:** Summary of advantages and disadvantages of classifcation systems.

Name of Classification	Year	Positive Aspects of Classification	Negative Aspects of Classification
Johnson and Strom [1]	1989	1. Original classification that classification systems are based upon 2. Establishedd a basic understanding/method of cummincation patholgy. More severe deformities/stiffness correspond to a greater stage 3. Recognition of tendon reactivity in early stages of AAFD/PCFD where no deformity can present with a reactive TP	1. No proven linearity of progression between stages 2. Fails to consider foot may not start in neutral 3. Focuses on the tibialis posterior as the prime driving force/causal 4. Stage 2 is very under simplified 5. Very little on the validation of the classification system
Myerson [2]	1997	1. Modified Johnson and Strom classification to establish deep deltoid failure in stage 4	1. Still focused on tibialis posterior as the prime cause 2. Failure to acknowledge that anteromedial ankle instability occurs prior to deep deltoid ligament failure 3. Assumed linearity of progression
Weinraub and Heilala [3]	2002	1. Understood that multiple factors determined the failure of the flatfoot 2. Recognized that the midtarsal joint played an important role in the stabilisation of flatfoot 3. Delinked deformity and tendon pathology	1. Still primarily focused upon the tibialis posterior tendon as the cause. 2. Based classification upon progressive inflammation/degenerative changes of the tendon
Bluman [4]	2007	1. Began to subclassify stage 2 and expand the different types 2. Graded level of deformity 3. Bluman classified a myriad of treatment options for all the subtypes	1. Classification based upon the tibialis posterior in early stages 2. Implies progression of deformity through set stages 3. No discussion of the spring ligament and other ligaments that fail
Deland [5]	2008	1. Recognition of the SL as a cause of potential instability	2. Still focused on the TP
Parsons [6]	2010	1. Began to subtype stage 2 into subtypes of A, B, C	1. Still focused on stage tibialis posterior as the cause of the flatfoot 2. Broadly based upon Johnson and Strom classification
Raikin [7]	2012	1. Previous classifications did not take into consideration the involvement of the mid-foot 2. Classification was based on anatomic location, including the ankle, hindfoot, and mid-foot 3. Subgroups based on characteristic clinical and radiographic findings 4. Treatment algorithms then suggested based on these findings	1. Several categories make communication more difficult 2. Still focused on tibialis posterior in early stages of the hindfoot
Pasapula [11]	2017	1. Introduced the concept of stage 0 2. Recognised that the tibialis posterior may or may not react despite the foot SL weakening and failing	Still used the Johnson and Strom classification Focused on the tibialis posterior in stage 1 Continued to simplify stage 2
Myerson [10]	2020	1. Readdresses the pathology away from the tibialis posterior tendon 2. Several categories allow a more accurate representation of deformity in the foot	1. Static weight-bearing imaging may miss or underestimate the associated dynamic instabilities 2. Multiple tendon reactivity may be a significant presentation and can present without deformity, which needs representation 3. Focusing on deformity detracts from representing feet with ligament instability and no deformity 4. 242 subtypes identified makes comparative analysis and communication of different grades and stages difficult

## Data Availability

No new data was created.
